# Unsupervised Clustering of Hyperspectral Paper Data Using t-SNE

**DOI:** 10.3390/jimaging6050029

**Published:** 2020-05-05

**Authors:** Binu Melit Devassy, Sony George, Peter Nussbaum

**Affiliations:** Department of Computer Science, Norwegian University of Science and Technology, 2802 Gjøvik, Norway; sony.george@ntnu.no (S.G.); peter.nussbaum@ntnu.no (P.N.)

**Keywords:** forensic document analysis, hyperspectral dimensionality reduction, forensic paper analysis, t-SNE, hyperspectral unsupervised clustering

## Abstract

For a suspected forgery that involves the falsification of a document or its contents, the investigator will primarily analyze the document’s paper and ink in order to establish the authenticity of the subject under investigation. As a non-destructive and contactless technique, Hyperspectral Imaging (HSI) is gaining popularity in the field of forensic document analysis. HSI returns more information compared to conventional three channel imaging systems due to the vast number of narrowband images recorded across the electromagnetic spectrum. As a result, HSI can provide better classification results. In this publication, we present results of an approach known as the t-Distributed Stochastic Neighbor Embedding (t-SNE) algorithm, which we have applied to HSI paper data analysis. Even though t-SNE has been widely accepted as a method for dimensionality reduction and visualization of high dimensional data, its usefulness has not yet been evaluated for the classification of paper data. In this research, we present a hyperspectral dataset of paper samples, and evaluate the clustering quality of the proposed method both visually and quantitatively. The t-SNE algorithm shows exceptional discrimination power when compared to traditional PCA with k-means clustering, in both visual and quantitative evaluations.

## 1. Introduction

Paper and ink are the two most important pieces of evidence in forensic document analysis; understanding the legibility of both of them has a vital role in the investigation of document forgery. To determine the originality of a document, forensic experts need to examine both paper and inks used. In the case of a multipage document, the presence of different paper types may lead to potential chances of forgery. In order to extract this information, the forensics experts rely on different techniques. The most commonly used techniques to detect forgeries includes ultraviolet (UV) and infrared (IR) imaging [1], chemical analysis and visual inspection [2]. The document analysts always prefer to use non-destructive methods, to preserve the original evidence even after the analysis. Unfortunately, chemical methods usually cause some damage to samples, and are therefore less popular compared to the non-destructive techniques. The major techniques that follow the non-destructive paradigm are; Fourier transform infrared (FTIR) [3], Raman spectroscopy [4], video spectral comparator (VSC) [5], multi-spectral imaging and hyperspectral imaging (HSI) [6,7].

**Figure 1:**
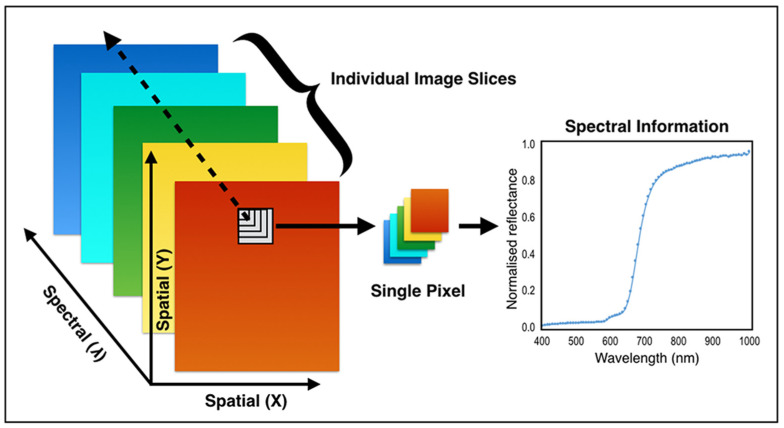
Hyperspectral image representation.

HSI combines spectroscopy and imaging in order to record the spectral information of the sample across the spatial area of interest. HSI captures hundreds of narrowband images in the visible and near infrared region, and this results in a large amount of data. Motivated by the possibility of non-destructive investigation of material properties, HSI has become one of the most popular and trustworthy tool for analysis in many fields of science, including food quality inspection [8], medical imaging [9], material science [10], cultural heritage imaging [11] and forensics investigation [12]. Compared to traditional RGB images, HSI images can be considered as three-dimensional data, with the third dimension encoding the spectral range as shown in Figure 1. Each pixel of HSI data represents the spectrum in that spatial point, and this information can be used as a material fingerprint for characterizing each point.

The HSI data contains redundant information, and it requires an efficient method to extract the most interesting and useful information [13]. When considering hyperspectral dimensionality reduction, a favorite method is the well-known PCA approach [14], as outlined in several papers [3,15,16]. Other traditional techniques such as Independent Component Analysis (ICA) [17] and Linear Discriminant Analysis (LDA) [18] as well as statistical methods [19,20,21]. Aside from the methods discussed, a new method known as t-Distributed Stochastic Neighbor Embedding (t-SNE) [22] is gaining popularity in dimensionality reduction related problems. t-SNE dimensionality reduction techniques are already deployed in HSI processing, and have obtained better results than the traditional methods. However, this technique has not yet been evaluated in HSI data of paper, hence we have decided to explore the power of t-SNE algorithm in the dimensionality reduction and visualization of hyperspectral data of paper samples. The main contribution of this research will be to test and evaluate the t-SNE based workflow for unsupervised clustering of HSI images of paper samples, and benchmarking the proposed method against PCA. To implement this, we have created an HSI dataset of 40 different paper samples.

t-SNE was chosen as a candidate because of the following advantages over the conventional methods. Primarily, t-SNE is one among the few algorithms that is capable of simultaneously retaining both local and global structure of the data; also, it calculates the probability similarity for points in high dimensional space as well as in low dimensional space. Since its invention, t-SNE has been introduced into many fields. We present a few of them here in order to show the range of applications. Walid et al. identified that t-SNE has better capability to resolve the bio-molecular intra-tumor heterogeneity from mass spectroscopy images [23]. Erdogan et al. applied t-SNE on the visualization of human tissue relationships [24], whilst in another study t-SNE was used as a scalable alternative to create visualizations (projections) enabling insight into the structure of time dependent data sets [25]. Another example is the report made by Kunihiko et al. which suggests visualizing curricula using a combination of cosine similarity, t-SNE, and scatter plots to help students select their courses [26]. In addition, Chen et al. found that the t-SNE algorithm can be used to optimize underwater target radiated noise spectrum features for the purpose of improving the accuracy and efficiency of the classification algorithm [27]. A few experiments touched upon the t-SNE of HSI data sets. One amongst them is made by Pouyet et al. [28] which uses t-SNE to visualize HSI data of paint pigments. Song et al. also demonstrated the capability of t-SNE for remote sensing data processing [29]. In addition, there are a few reports which are not focused on dimensionality reduction, but which nevertheless utilize t-SNE and HSI data [30,31].

Performance of the proposed method is evaluated against PCA [14], which is identified as one of the most commonly used methods for dimensionality reduction. As well as visual comparison and quantitative methods are also used to get the clustering quality of processed data from both methods by using k-means clustering.

The remaining part of this paper is organized into three parts; the first part will explain the HSI acquisition, sample preparation, algorithms and evaluation methods; the following part will discuss results; and the paper ends with a conclusion that points to possible future works.

## 2. Materials and Methods

### 2.1. Hyperspectral Acquisition

**Figure 2:**
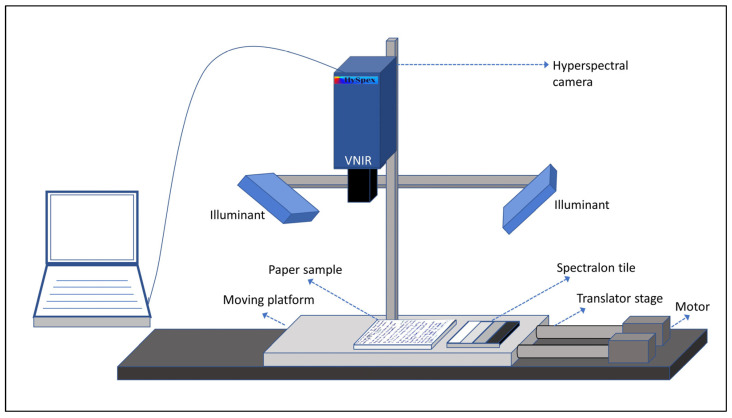
Hyperspectral acquisition setup.

The acquisition setup used here is similar to this experiment [32], which used to capture HSI data of inks. Hyperspectral image acquisition of the paper samples is performed using a push-broom hyperspectral camera HySpex VNIR-1800 [33]. The VNIR-1800’s wavelength range is from 400 nm to 1000 nm, with a spectral sampling of 3.18 nm and a spatial resolution of 1800 pixels across the field of view that captures 10 cm in width at the face of the chart. The acquisition setup is shown in Figure 2, where the chart containing different paper samples is placed on a moving translator with the camera positioned perpendicular to the translator stage. The samples are illuminated by two halogen light sources in 45°:0° geometry with respect to the camera. Pre-processing software HySpex RAD performs the basic camera corrections, such as dark current subtraction, sensor corrections, and radiometric calibration. Following calibration, the software converts the acquired raw images into the sensor absolute radiance values.

A Contrast Multi-Step Target [34] with known reflectance values is used as a reference, and is present in the scene. This reference target is used in order to recover the reflectance of the paper samples.

### 2.2. Samples and Data

**Figure 3:**
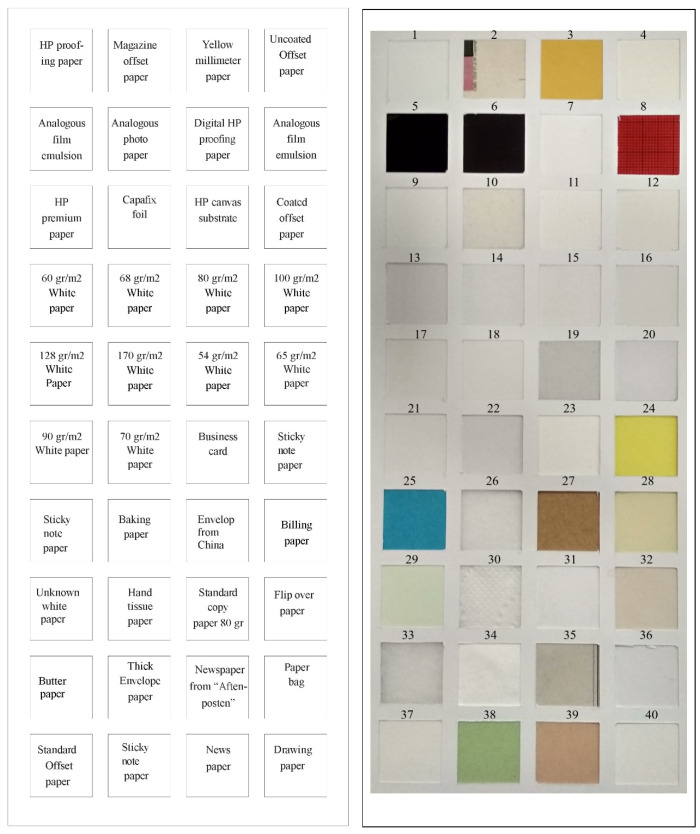
Paper checker. The left-hand side describes the types of paper, with the corresponding sample on the right-hand side. Identification numbers are marked above each sample.

Samples from 40 commonly used paper types are collected and randomly arranged as a checkerboard pattern as shown in Figure 3. The paper samples are arranged in a grid format of ten rows and four columns, where each sample is a square shape with 4-cm long sides. Since it looks similar to a standard color checker, we called it a “paper checker”. To ensure a more generic data set, the paper checker was prepared with papers of different colors, thicknesses, age, purpose and from different manufacturers.

Hyperspectral images of the paper checker are captured using the hyperspectral camera, and data pre-processing is performed with associated software. The acquired HSI data cube has a dimension of 1800 x 7500 x 186, where 1800 x 7500 is the spatial resolution and 186 indicates the number of spectral bands. A standard reference target (Contrast Multi-Step Target) with known reflectance is captured along with the paper samples and this information is later used to calculate the normalized reflectance of the samples.

To process the t-SNE and PCA, we use different sample areas (from 25 to 2500 pixels in total) from each paper type, and tuned for better perplexity (an input parameter of t-SNE algorithm). The sample areas are selected using squares of regions of interest (ROIs) around the center point of each paper sample by varying the side length of ROI from five to fifty.

### 2.3. t-Distributed Stochastic Neighbor Embedding (t-SNE)

In 2008, Van der Maaten and Hinton [22] introduced the t-SNE algorithm as an innovative tool for the scaling down of the multidimensional data. This technique gradually gained acceptance in the machine learning community due to its remarkable ability to scale high dimensional data to lower dimensions. Initially, the algorithm converts high-dimensional Euclidean distances between data points into conditional probabilities that represent similarities, by applying SNE (Stochastic Neighbor Embedding) to the data points. The similarity of data point xj to data point xi is expressed by the conditional probability Pj|i, defined as in the equation below

(1) $$P_{j|i}=\frac{\exp\left(\;\frac{{-||x_{i}-x_{j}||}^{2}}{2\sigma_{i}^{2}}\right)}{\sum_{k\neq i}\exp\left(\frac{{-||x_{i}-x_{k}||}^{2}}{2\sigma_{i}^{2}}\right)}$$

Then the probabilities in the original space are defined as shown in the equation below

(2) $$P_{i,j\;}=\frac{\left(P_{i|j}+P_{j|i}\right)}{2n}$$

where *n* is the size of the data set. The smoothness measure of the effective number of neighbors is called “perplexity”, which is an input parameter to the t-SNE algorithm, and it can be defined as below.

(3) $$Perp\left(P_{i}\right)=2^{H\left(P_{i}\right)}$$

where H(P_i_) is the Shannon entropy P_i_ measured in bits.

(4) $$H\left(P_{i}\right)=-\underset{j}{\sum }P_{j|i\;}\log_{2}P_{j|i}$$

Based on the pairwise distances of the points, this method automatically determines the variance σ_i_, such that the effective number of neighbors coincides with the user provided perplexity [22]. The t-SNE uses the Student t-Distribution with a single degree of freedom, to avoid overcrowding. Using this distribution, the probability at low dimension q_ij_, can be defined as shown in the equation below.

(5) $$q_{ij\;}=\frac{{\left(1+{\left||y_{i}-y_{j}|\right|}^{2}\right)}^{-1}}{\sum_{k\neq l}{\left(1+{\left||y_{k}-y_{l}|\right|}^{2}\right)}^{-1}}$$

The t-SNE algorithm then uses the Kullback–Leibler divergence [35] together with a gradient-based technique to find the projections of the input data x_i_ in lower dimension as y_i_.

### 2.4. Principal Component Analysis (PCA)

In this experiment, we consider PCA as a standard reference for comparison of clustering quality because PCA is arguably the tool most extensively used for dimensionality reduction [14] and is referred to in several scientific papers within different domains. PCA is a multivariate analysis technique used to extract important information from the data into a set of new orthogonal variables called principal components. PCA identifies patterns in data, and can express the data in such a way as to highlight their similarities and differences [36]. Whilst it is hard to identify the patterns in high dimensional data because it is difficult to visualize, PCA can solve this problem by mapping the high dimensional data into lower dimensions.

### 2.5. Clustering Performance Evaluation

To measure clustering performance, we use four well-known clustering indices; these are Silhouette Index, Normalized Mutual Information, Homogeneity Index (HI) and Completeness Index (CI). These methods are used to evaluate the clusters produced by the k-means clustering algorithm [37] from PCA or t-SNE processed data.

The *Silhouette Index* (SI) [38] defines how indistinguishable an object is from its own cluster (tightness) with respect to other clusters (separation). An SI value of 1.0 indicates a perfect clustering value, whilst -1.0 indicates the poorest clustering, and values near 0.0 indicate intersecting clusters. *Normalized Mutual Information* (NMI) [39] gives an estimate of the overlap between clusters. An NMI value of 1.0 indicates perfect clustering, whilst 0.0 indicates a poor clustering with respect to the given labels. *Homogeneity Index* (HI) [40] verifies whether each cluster contains only the data points from a single class or not. The *Completeness Index* (CI) [40] score indicates whether or not all data points that have the same labels are assigned to the same cluster. HI and CI scores can vary between 0.0 and 1.0, with better clustering yielding higher values.

### 2.6. Data Processing

**Figure 4:**
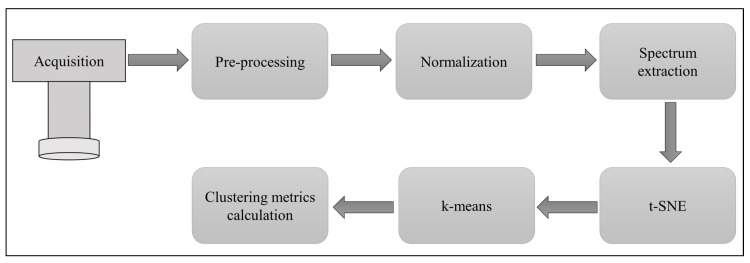
Data processing pipeline for the proposed method.

The processing pipeline is illustrated in Figure 4. The hyperspectral camera performs the acquisition, a pre-processing (non-uniformity and dark offset correction of image data) is then done using the camera software. The next block represents the normalization performed using the data from the reference target. From the normalized reflectance data, a spectrum corresponding to each pixel can be extracted using the coordinate positions. The collected spectra are then sent to PCA or t-SNE algorithms for dimensionality reduction, followed by the k-means algorithm. In the final block, we calculate the clustering quality matrices from the k-means results using the known labels.

**Figure 5:**
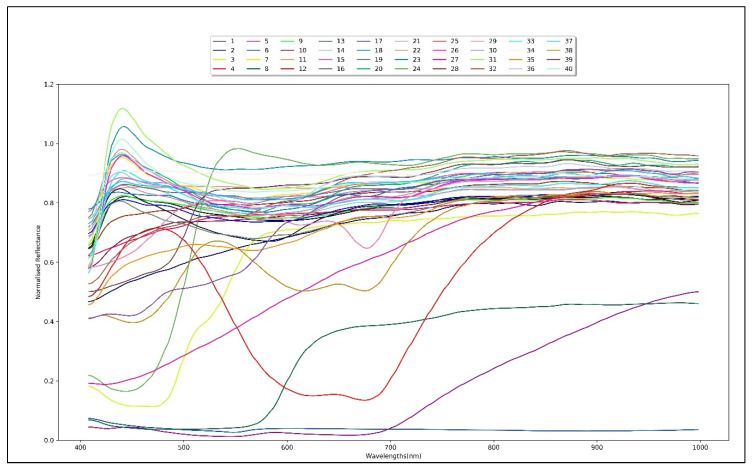
Average normalized reflectance spectrum of 40 paper samples.

Figure 5 illustrates the average normalized reflectances for the 40 samples used in this experiment.

## 3. Results and Discussions

This experiment used 40 papers samples, and selected different spectral sample sizes between 25 and 2500 pixels from each paper samples, also tuned for perplexity. The clustering indices are measured 20 times for each combination of sample count and perplexity, and the average classification indices obtained for optimal perplexities are given in Table 1 below.

The NMI value for the data from t-SNE obtained a high score (0.92) indicating a good clustering, compared to 0.72 for the PCA processed data. The CI and HI indices of clustering obtained from t-SNE processed data also achieved a score close to unity, demonstrating the efficiency of t-SNE dimensionality reduction compared to PCA. Finally, the SI index, which indicates the tightness of the clustering, gives t-SNE algorithm an upper hand over PCA.

**Figure 6:**
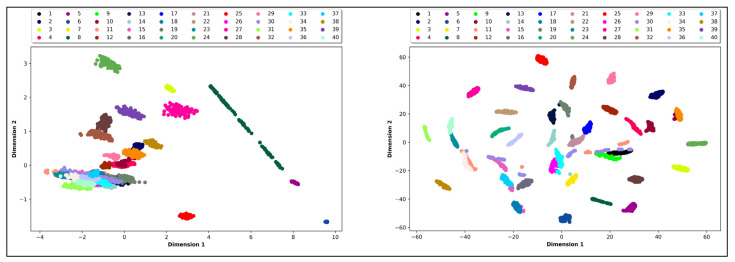
Clustering results of 40 paper samples, with a sample size of 100 spectra. Left-hand plot is obtained using Principal Component Analysis (PCA), and the right-hand plot is obtained using t-Distributed Stochastic Neighbor Embedding (t-SNE).

Figure 6 visualizes the results obtained from dimensionality reduction from PCA and t-SNE (where the original spectral dimension of 186 bands has been reduced to two-dimensional data). A simple visual inspection is enough to conclude that the t-SNE clusters are more distinguishable than those of PCA. In this context, t-SNE provides a better visualization than PCA, and this helps us to predict the nature of the data.

From the clustering indices and visual inspection, it is clear that for HSI data of paper samples the t-SNE algorithm surpasses the results obtained from PCA. These findings are not surprising [32], since PCA always tries to find a linear relationship between data points, and this may fail at many data points while dealing with highly non-linear data such as a spectrum with 186 dimensions. This is because PCA projects the data (n-dimensional) onto an m-dimensional (m < n) linear subspace defined by the leading eigenvectors of the original data’s covariance matrix, to obtain a global linear model [41]. However, t-SNE is designed to mitigate this problem by extracting non-linear relationships, which helps t-SNE to produce a better classification.

**Figure 7:**
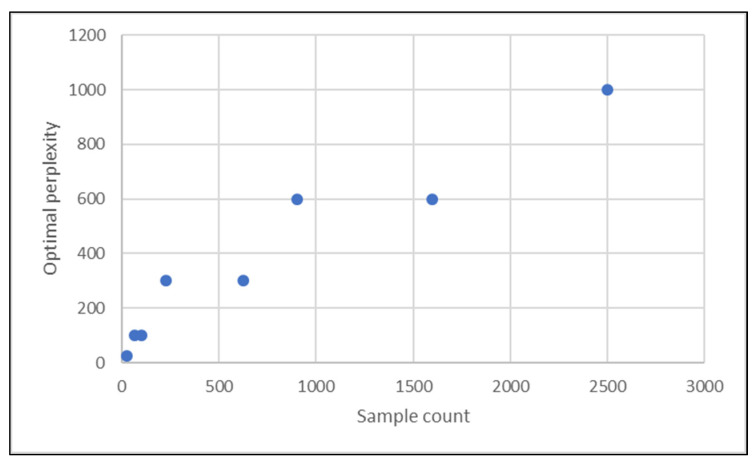
Optimal perplexity relation against sample count.

The experiment uses different sample sizes of between 25 and 2500 pixels, and for each sample size the t-SNE is executed over a list of perplexities in order to find the optimal perplexity. The list of perplexities used are 5, 10, 25, 50, 100, 300, 600 and 1000, and we select as our optimal perplexity value that which gives the highest value for all clustering quality parameters. Table 2 lists the sample counts used and the optimal perplexity values obtained, along with the clustering index values corresponding to the optimal perplexity. It is observed that the optimal perplexity value depends on the sample size, which is visualized in Figure 7.

**Figure 8:**
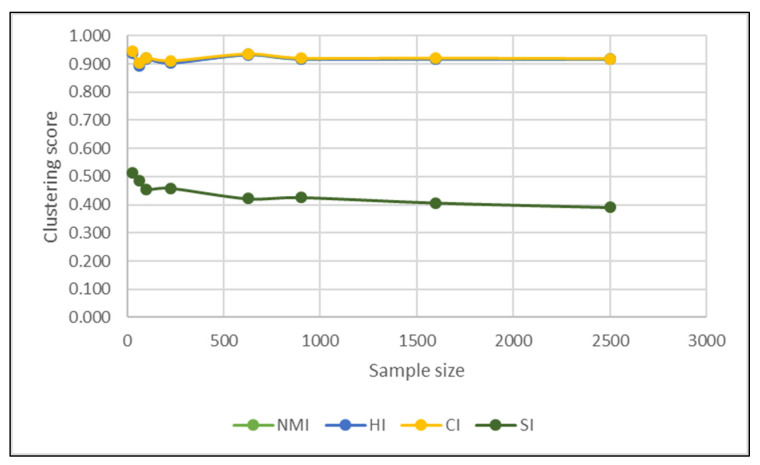
Impact of sample size over the clustering index for the t-SNE algorithm.

A more detailed analysis of results leads us to the important finding that when using t-SNE the clustering indices are quite stable at varying clustering sizes, as seen in Table 2 and visualized in Figure 8. This demonstrates that the sample size has little influence on the dimensionality reduction power of the t-SNE algorithm.

**Figure 9:**
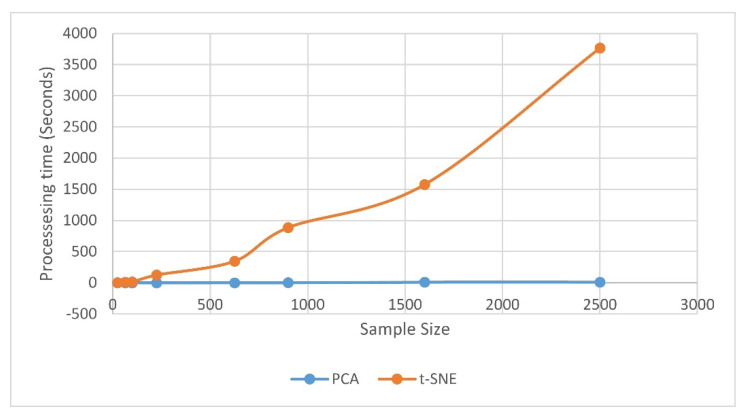
Sample size impact on the processing time.

The t-SNE algorithm does require more computation time because of its quadratic time complexity; compared to PCA this might be the major disadvantage of t-SNE. In the present study, for 2500 samples from 40 different papers, t-SNE consumes 3763.3 seconds on average while PCA consumes 10.2 seconds. The performance is measured for an Intel Core i7 8650U CPU with 16 GB of RAM, and Figure 9 shows the variation in time consumption against sample size.

While processing with t-SNE, the parameter perplexity needs to be optimized for those particular data, compared to the straightforward processing of PCA. This parameter tuning introduces extra processing into the workflow, which is not required for PCA.

## 4. Conclusions and Future Work

The proposed unsupervised clustering workflow using the t-SNE dimensionality reduction technique was applied to our HSI paper data set. The clustering quality was compared to the PCA results, and it was shown that the proposed method outperformed the PCA. An HSI database of paper samples containing forty different paper types was created as a part of this work. In addition, we executed the perplexity tuning, and compared the computational expenses between PCA and t-SNE. It can be concluded that the non-linear dimensionality reduction method is suitable for paper spectral data, which is non-linear in nature. This non-linear method for clustering paper spectral data should ideally be validated against a number of other non-linear methods, which may be considered for a follow-up to this work.

## Figures and Tables

**Table 1:** Average values of clustering indices.

Validation Indices	PCA	t-SNE
NMI	0.72	0.92
HI	0.70	0.92
CI	0.75	0.92
SI	0.34	0.44

**Table 2:** Results obtained for different sample sizes and optimal perplexity.

Sample Count	25		64		100		225		625		900		1600		2500	
Optimal Perplexity	25		100		100		300		300		600		600		1000	
	PCA	t-SNE	PCA	t-SNE	PCA	t-SNE	PCA	t-SNE	PCA	t-SNE	PCA	t-SNE	PCA	t-SNE	PCA	t-SNE
NMI	0.78	**0.94**	0.76	**0.90**	0.74	**0.92**	0.73	**0.91**	0.70	**0.93**	0.70	**0.92**	0.69	**0.92**	0.69	**0.92**
HI	0.75	**0.94**	0.73	**0.90**	0.71	**0.92**	0.71	**0.90**	0.67	**0.93**	0.68	**0.92**	0.67	**0.92**	0.67	**0.92**
CI	0.81	**0.94**	0.79	**0.91**	0.76	**0.92**	0.75	**0.91**	0.72	**0.94**	0.73	**0.92**	0.71	**0.92**	0.72	**0.92**
SI	0.39	**0.51**	0.38	**0.48**	0.37	**0.46**	0.34	**0.46**	0.31	**0.42**	0.33	**0.43**	0.29	**0.41**	0.31	**0.39**
